# Leptin and insulin stimulation of signalling pathways in arcuate nucleus neurones: PI3K dependent actin reorganization and K_ATP _channel activation

**DOI:** 10.1186/1471-2202-5-54

**Published:** 2004-12-06

**Authors:** Shirin Mirshamsi, Hilary A Laidlaw, Ke Ning, Erin Anderson, Laura A Burgess, Alexander Gray, Calum Sutherland, Michael LJ Ashford

**Affiliations:** 1 Division of Pathology & Neuroscience, Ninewells Hospital & Medical School, University of Dundee, Dundee DD1 9SY UK; 2 Division of Signal Transduction, School of Life Sciences, University of Dundee, Dundee DD1 5EH UK

## Abstract

**Background:**

Leptin and insulin are long-term regulators of body weight. They act in hypothalamic centres to modulate the function of specific neuronal subtypes, by altering transcriptional control of releasable peptides and by modifying neuronal electrical activity. A key cellular signalling intermediate, implicated in control of food intake by these hormones, is the enzyme phosphoinositide 3-kinase. In this study we have explored further the linkage between this enzyme and other cellular mediators of leptin and insulin action on rat arcuate nucleus neurones and the mouse hypothalamic cell line, GT1-7.

**Results:**

Leptin and insulin increased the levels of various phosphorylated signalling intermediates, associated with the JAK2-STAT3, MAPK and PI3K cascades in the arcuate nucleus. Inhibitors of PI3K were shown to reduce the hormone driven phosphorylation through the PI3K and MAPK pathways. Using isolated arcuate neurones, leptin and insulin were demonstrated to increase the activity of K_ATP _channels in a PI3K dependent manner, and to increase levels of PtdIns(3,4,5)P_3_. K_ATP _activation by these hormones in arcuate neurones was also sensitive to the presence of the actin filament stabilising toxin, jasplakinolide. Using confocal imaging of fluorescently labelled actin and direct analysis of G- and F-actin concentration in GT1-7 cells, leptin was demonstrated directly to induce a re-organization of cellular actin, by increasing levels of globular actin at the expense of filamentous actin in a PI3-kinase dependent manner. Leptin stimulated PI3-kinase activity in GT1-7 cells and an increase in PtdIns(3,4,5)P_3 _could be detected, which was prevented by PI3K inhibitors.

**Conclusions:**

Leptin and insulin mediated phosphorylation of cellular signalling intermediates and of K_ATP _channel activation in arcuate neurones is sensitive to PI3K inhibition, thus strengthening further the likely importance of this enzyme in leptin and insulin mediated energy homeostasis control. The sensitivity of leptin and insulin stimulation of K_ATP _channel opening in arcuate neurones to jasplakinolide indicates that cytoskeletal remodelling may be an important contributor to the cellular signalling mechanisms of these hormones in hypothalamic neurones. This hypothesis is reinforced by the finding that leptin induces actin filament depolymerization, in a PI3K dependent manner in a mouse hypothalamic cell line.

## Background

Leptin and insulin function as peripherally-derived hormone signals involved in the long-term regulation of energy balance [1-4]. Their circulating levels are directly proportional to adipose mass and CNS access occurs via saturable receptor-mediated processes. The primary CNS target for these adipostats is the ARC, where leptin and insulin receptors are highly expressed, and where direct administration of either hormone has a potent effect on food intake and body weight. Two specific ARC neurone populations have been strongly implicated in sensing changes in levels of circulating leptin and insulin and transducing these signals into neuronal outputs [1,3]. These "first-order" neurones encompass the melanocortin precursor, POMC containing neurones and NPY and AgRP co-containing neurones, the former associated with catabolic, the latter anabolic, outputs. Leptin and insulin increase POMC mRNA levels and decrease NPY & AgRP mRNA levels respectively.

However, transcriptional control is not the only effector mechanism elicited by these hormones on ARC neurones. Electrophysiological studies have shown that leptin depolarizes and increases the firing rate of ARC POMC neurones and inhibits the tone of NPY/AgRP neurones [5]. Although the electrophysiological actions of insulin have not been reported for identified POMC and NPY/AgRP neurones, both leptin and insulin have been demonstrated to inhibit, by hyperpolarization, the firing of a sub-population of ARC neurones, identified by their sensitivity to changes in extracellular glucose concentration [6,7]. For these latter neurones, termed glucose-responsive (GR), K_ATP _channels have been identified as an effector mechanism through which leptin and insulin elicit neuronal inhibition. Consequently, leptin and insulin signal the status of body energy stores by activating their receptors on ARC neurones, eliciting changes in the electrical activity and amounts of releasable peptides in specific neuronal populations, leading to compensatory effector outputs, such as changes in food intake, energy balance and glucose homeostasis [8].

Obese humans have elevated leptin and insulin levels, indicative of central resistance to these hormones [9]. The mechanisms underlying this resistance are unclear, with defective hormone passage through the BBB and flawed receptor-signal transduction in ARC neurones being the prime candidates [10,11]. Consequently, it is important to understand the molecular mechanisms underlying leptin and insulin receptor modulation of ARC first-order neurones. Leptin and insulin, by stimulation of their respective receptors, have been demonstrated to activate various signalling pathways in peripheral tissues [10-13]. However, as these hormones induce seemingly identical actions on ARC neurones, both in terms of behavioural output and effects on ARC neurone excitability, some parallelism or convergence of signalling is likely [12,13]. Leptin, by binding to the long form of the leptin receptor (ObRb) has been demonstrated to activate three main signalling cascades, JAK2 – STAT3, MAPK and PI3K, the latter two of which are also intermediates in insulin receptor activation [14,15]. However, recent studies have strongly implicated PI3K as the key signalling intermediate in leptin and insulin actions on hypothalamic neurones influencing food intake and body weight [16,17].

Thus, to elucidate further the pathways that contribute to convergent actions of leptin and insulin on ARC neurones, we have examined the phosphorylation status of key leptin and insulin signalling intermediates in the ARC and have explored the linkage, with a focus on PI3K mediated signal transduction pathways, between these hormones and ARC neurone K_ATP _channel activation.

## Results

### Leptin and insulin stimulate phosphorylation of signalling proteins in ARC

**Figure 1:**
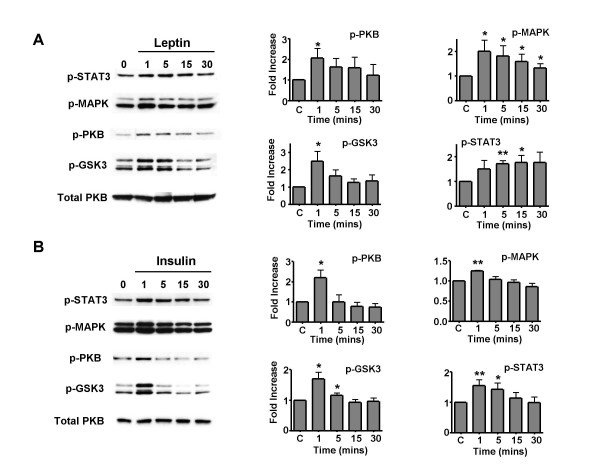
**Effects of leptin and insulin on phosphorylation of STAT3, MAPK, PKB and GSK3 **Rat ARC wedges were incubated for 0, 1, 5, 15 or 30 minutes with 10 nM leptin (A) or 0.1 – 1 nM insulin (B) before cells were lysed and equal amounts of lysate were subjected to SDS-PAGE and transferred to nitrocellulose membrane. The phosphorylated levels of p42/p44 MAPK, PKB, STAT3 and GSK3α/β were detected by immunoblotting with appropriate specific antibodies. The total amount of PKB is also shown. Bands were quantified using densitometry. The values are expressed as relative to the corresponding aCSF control group, and normalized for protein loading. Values represent the mean ± SEM for between 4–6 animals for each time point. * P < 0.05 and ** P < 0.01.

**Figure 2:**
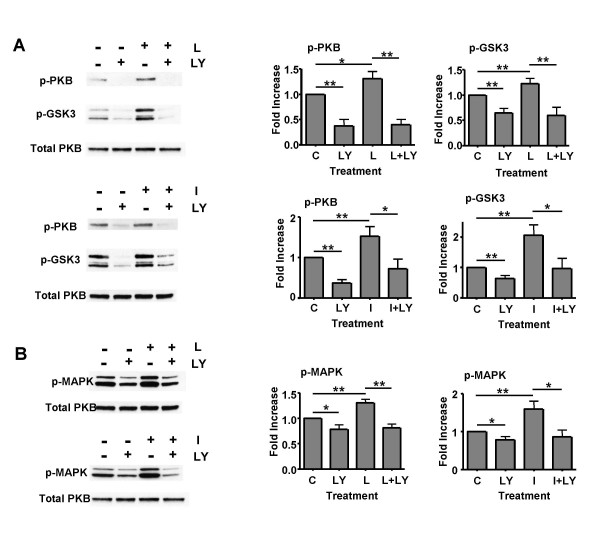
**Changes in phosphorylation of PKB, GSK3 and MAPK by inhibition of PI3K **Rat ARC wedges were pretreated with 10 μM LY294002 or aCSF for 20 minutes and incubated for 1 minute with 10 nM leptin or 1 nM insulin or aCSF. Equal amounts of protein lysate were subjected to SDS-PAGE and transferred to nitrocellulose membrane. The phosphorylated levels of PKB, and GSK3αβ (A) and p42/p44 MAPK (B) were detected by immunoblotting with appropriate specific antibodies. The total amount of PKB is also shown. Bands were quantified using a densitometer. The values are expressed as relative to the corresponding aCSF control group, and normalized for protein loading. Values represent the mean ± SEM for between 4–5 and 3–5 animals for each time point with leptin and insulin respectively. * P < 0.05 and ** P < 0.01.

Rat hypothalamic tissue sections, predominantly made up of arcuate nucleus, were treated with aCSF alone or with leptin (10 nM) or insulin (0.1 or 1 nM, which produced identical results) for 1, 5, 15 and 30 minutes. Leptin and insulin stimulation induced comparable immunoblot profiles (Figure 1), with increased levels of phosphorylated STAT3 (p-STAT3), phosphorylated MAPK (p-MAPK), phosphorylated PKB/Akt (p-PKB) and GSK3 (p-GSK3). The phosphorylation status of the latter two proteins, PKB and its downstream effector GSK3, were utilised as a sensitive assay for hormone activation of PI3K. Leptin (10 nM) or insulin (0.1 nM) treatment was observed to cause an increase in phosphorylation of all four proteins. This increase in phosphorylation was generally transient with the highest levels of phosphorylation at the 1 and/or 5 minute time points. Subsequent to this peak level, in the majority of experiments, the phosphorylation was not sustained over the time period examined and returned to control values within 30 minutes (Figure 1A,1B). These data demonstrate that all 3 pathways potentially contribute to insulin and leptin signalling in ARC neurones and thus play a role in connecting leptin or insulin receptor activation to neuronal effector outputs. As both leptin and insulin signalling in the ARC require PI3K activity for reduction in food intake and body weight [16,17], we examined the sensitivity of the phosphorylation of PKB/GSK3 and MAPK to the presence of PI3K inhibitors. Isolated ARC sections were incubated either in control aCSF, 10 nM wortmannin or 10 μM LY294002, for 20 minutes prior to exposure to control aCSF, leptin (10 nM) or insulin (0.1 nM), in the continued presence of the appropriate inhibitor. The presence of LY294002 prevented leptin or insulin induced phosphorylation of PKB and GSK3 following 1 minute exposure to these hormones, illustrated in Figure 2A, as expected for proteins downstream of PI3K [18]. Furthermore, the presence of the PI3K inhibitor *per se *reduced p-PKB and p-GSK3 levels significantly, indicating that PI3K is active to a limited degree in these ARC neurones. Similar results were obtained for wortmannin (data not shown). However, surprisingly the PI3K inhibitors also reduced the leptin- and insulin-stimulated phosphorylation of MAPK (Figure 2B). These data further establish PI3K as a key component of neuronal leptin and insulin signalling in ARC neurones and suggest a potential role for PI3K in leptin and insulin driven transcriptional activity. Because the phosphorylation status of these signalling intermediates was examined in whole ARC extracts, this supplies little information as to the mechanisms by which adiposity hormones target specific ARC neurones. Thus, we have tried to delineate the molecular events connecting leptin and insulin receptor activation, PI3K activity and effector outputs. Here we focus on the activation of K_ATP _channels, responsible for leptin and insulin inhibition of an electrophysiologically identified subset of ARC neurones [6,7].

### Leptin and insulin activate K_ATP _channels in acutely isolated ARC neurones

**Figure 3:**
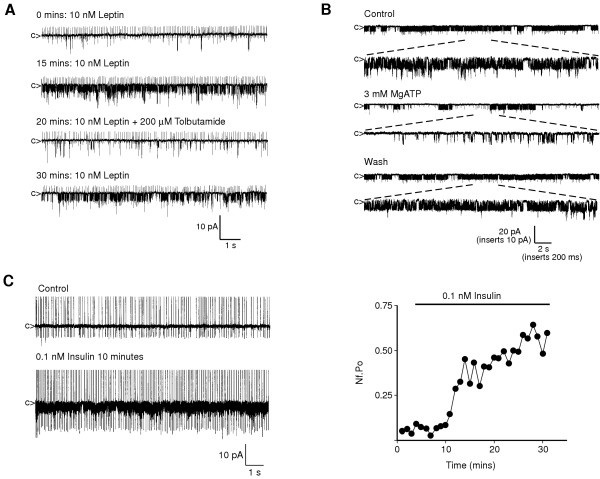
**Leptin and insulin activate large conductance K_ATP _in ARC neurones ***A*, representative cell-attached recording from an acutely dissociated ARC neurone. Leptin (10 nM), present in the electrode solution, increased the activity of a K^+ ^channel, which was inhibited reversibly by bath application of 200 μM tolbutamide. Upward deflections in this and subsequent cell-attached recordings are extracellularly recorded action current activity. *B*, representative recording from an inside-out patch under asymmetrical K^+ ^conditions and held at 0 mV obtained from an acutely dispersed ARC neurone, following cell-attached leptin-induced increase in channel activity. Note that bath application of 3 mM MgATP reversibly inhibited K^+ ^channel activity. Inserts are expanded regions of traces showing channel activity in more detail. *C*, representative cell-attached recording from an acutely dissociated ARC neurone. Under control conditions, few channel openings are observed. Subsequent to bath application of 0.1 nM insulin, there is a marked increase in K_ATP _channel activity. Corresponding diary plot of channel activity (N_f_.P_o_) with time displays the insulin induced increase in activity.

Cell-attached recordings from rat isolated ARC neurones were used to confirm that leptin and insulin activate the large conductance K_ATP _channel as previously described [6,7]. Leptin, present in the recording electrode during cell-attached recordings, increased mean K^+ ^channel activity in 45% of unidentified neurones (n = 25/55). Mean channel activity (N_f_.P_o_), 1–2 minutes following cell attached formation was 0.08 ± 0.02 and increased to 0.38 ± 0.03 (n = 10, P < 0.01) after peak activation had occurred (10.6 ± 1.0 minutes after patch formation). In control cell-attached recordings of between 10–25 minutes, with 10 nM leptin in the pipette solution, there was no effect on K^+ ^channel currents observed in other ARC neurones (n = 30). During peak K_ATP _channel activation by leptin, bath application of the K_ATP _channel inhibitor tolbutamide (200 μM), reduced mean channel activity by 56 ± 12% (n = 4; P < 0.05), an effect reversible on washout of drug (Figure 3A). Following leptin-induced increase in channel activity, patch excision into the inside-out configuration allowed channel sensitivity to ATP to be assessed. At a patch potential of 0 mV and in asymmetric cation gradients, mean channel activity was 0.40 ± 0.09 (n = 3) and bath application of 3 mM MgATP (Figure 3B) reversibly reduced channel activity by 67.1 ± 9.7% (P < 0.05). Current-voltage relations under symmetrical K^+ ^conditions were linear, with a mean single channel conductance of 156 ± 15 pS (n = 3). The sensitivity to tolbutamide, ATP and single channel characteristics are consistent with leptin activation of the large conductance K_ATP _channel of GR neurones [6,19]. In a separate series of cell-attached recordings from isolated ARC neurones, bath application of insulin (0.1 – 10 nM) also increased K_ATP _channel activity in 45% of unidentified neurones (n = 14/31). N_f_.P_o _increased from 0.14 ± 0.03 under control conditions to 0.49 ± 0.08 (n = 7; P < 0.01) after approximately 10 – 20 minutes exposure to insulin (Figure 3C). Insulin had no effect on other K^+ ^channel currents in cell-attached recordings from the remaining neurones (n = 17). Consequently, these data are in agreement with previous studies on rat ARC GR neurones [6,7].

### Leptin and insulin activation of ARC neurone K_ATP _is PI3K dependent

**Figure 4:**
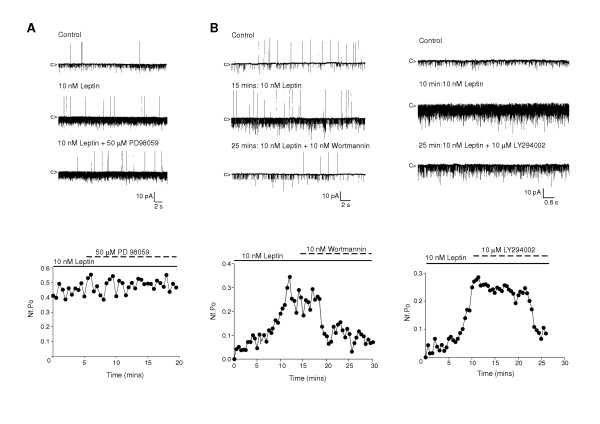
**PI3K activity mediates leptin activation of K_ATP _**Representative cell-attached recordings with leptin (10 nM), present in the recording electrode. *A*, traces illustrate that leptin-induced increase in K_ATP _activity is not reversed on bath application of the MEK inhibitor, PD 98059 (50 μM). The corresponding diary plot for part of the recording, initiated 20 mins after recording began, and following attainment of maximal leptin-induced K_ATP _channel activity is shown. *B*, traces show that leptin-induced K_ATP _channel activity is inhibited by subsequent application of the PI3K inhibitors, wortmannin (10 nM) or LY294002 (10 μM). Corresponding diary plots for N_f_.P_o _from a single experiment for each PI3K inhibitor are shown below the relevant traces.

As leptin and insulin activation of K_ATP _channels in ARC neurones is rapid (<5–10 minutes) and the leptin increase in K_ATP _activity demonstrated in isolated patches [6], this action is unlikely to be mediated by changes in transcription. Furthermore, in cell-attached recordings, following leptin (10 nM) stimulated K_ATP _channel activity, application of the MAPK pathway inhibitor, PD98059 (10 μM; n = 4) had no effect on N_f_.P_o _(Figure 4A). A previous study has demonstrated that insulin activated ARC neurone K_ATP _channels are similarly insensitive to this MAPK pathway inhibitor [7]. However, inhibition of PI3K does reverse both leptin (Figure 4B) and insulin-induced activation of ARC neurone K_ATP _channels. Leptin (10 nM) increased K_ATP _mean N_f_.P_o _from 0.21 ± 0.10 to 0.68 ± 0.28 (n = 3, P < 0.02), and subsequent bath application of 10 nM wortmannin reduced K_ATP _activity to a mean value of 0.33 ± 0.13 (n = 3, P < 0.02) over a period of 15–20 minutes, an N_f_.P_o _indistinguishable from control (P > 0.4). Similarly, in a separate series, leptin increased N_f_.P_o _from 0.15 ± 0.04 to 0.35 ± 0.05 (n = 4, P < 0.05) and subsequent application of 10 μM LY294002 reduced N_f_.P_o _to 0.19 ± 0.04 (n = 4, P < 0.01) within 15–20 minutes. Essentially identical data have been reported previously for the effects of these PI3K inhibitors on insulin-activated ARC K_ATP _channel activity [7]. Thus these results demonstrate that leptin and insulin signalling pathways converge on PI3K to elicit GR neurone hyperpolarization, and confirm that PI3K is a key enzyme in individual ARC neurone responsiveness to both leptin and insulin.

**Figure 5:**
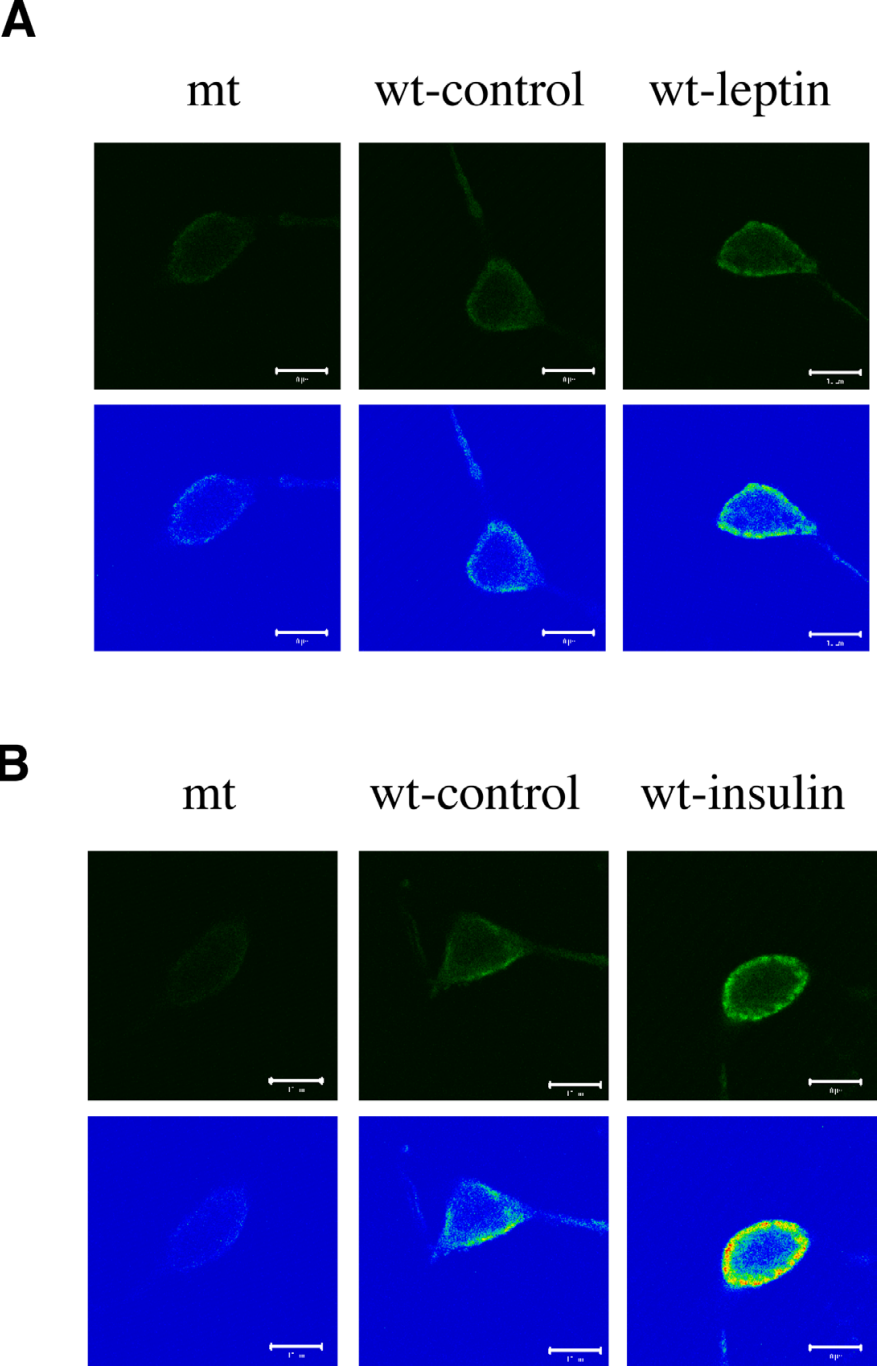
**Leptin and insulin increase PtdIns(3,4,5)P_3 _in isolated neurones **Acutely isolated ARC neurones were incubated in the absence and presence of 10 nM leptin (A) or 1 nM insulin (B) for 10 minutes. Cells were fixed and permeabilized, as described in Methods, prior to incubation with wild type (wt) or K273A mutant (mt) PH-GRP1-GFP fusion protein for 1 hour. Cells were subsequently processed for visualising GFP by confocal microscopy. Note that leptin and insulin increased the binding of wild type PH-GRP1-GFP in ARC neurones, and this is shown as both the fluorescence image (upper panels in A, B) and as a false colour image (lower panels in A, B), where blue represents low or non-detectable fluorescence and red the highest fluorescence intensity.

Such a central role for PI3K suggests that its main lipid product, PtdIns(3,4,5)P_3 _may serve as an important second messenger for downstream effectors such as the K_ATP _channel. The mechanism by which PtdIns(3,4,5)P_3 _recognises downstream target proteins is by binding to specialised phosphatidylinositol recognition sites, such as the pleckstrin homology (PH) domain [20]. Thus, to demonstrate that PtdIns(3,4,5)P_3 _production is elevated in ARC neurones following exposure to leptin and insulin, we used the PH domain of GRP-1, which selectively binds PtdIns(3,4,5)P_3 _[21] coupled to GFP (PH-GRP1-GFP) in an overlay assay on fixed freshly isolated ARC neurones. In non-stimulated ARC neurones there is significant labelling of all neurones with PH-GRP1-GFP (Figure 5). This is likely due to inherent PI3K activity of the neurones, rather than non-specific binding, as a PH-GRP1-GFP fusion protein with a single point mutation (K273A), which does not bind PtdIns(3,4,5)P_3 _[21,22], displays very little reactivity with non-stimulated ARC neurones (n = 7). Stimulation of isolated ARC neurones with leptin (10 nM) for 10 minutes resulted in a proportion (38 ± 8%; n = 5) of dispersed neurones displaying increased fluorescence after treatment with wild type fusion protein (Figure 5A). In addition, exposure of dispersed neurones to insulin (1 nM) for 5–10 minutes induced increased binding of PH-GRP1-GFP fusion protein in a similar proportion of neurones (43 ± 15%; n = 6), although insulin appeared to induce greater levels of binding/fluorescence than leptin (Figure 5B). However, leptin and insulin driven phosphorylation of PKB and GSK3 along with induction of elevated PtdIns(3,4,5)P_3 _levels in ARC neurones, are only indicative of increased PI3K activity. Thus we examined IRS-2 associated PI3K activity [16] in isolated ARC wedges exposed for one or two minutes to leptin (up to 50 nM) or insulin (up to 100 nM). Although we observed an increase in activity in 4/8 and 4/7 experiments for leptin and insulin respectively, there was no overall significant increase observed (data not shown). This may be due to the relative paucity of leptin and /or insulin sensitive neurones in the overall cellular population.

### Leptin and insulin activation of GR neurone K_ATP _requires actin filament re-organisation

**Figure 6:**
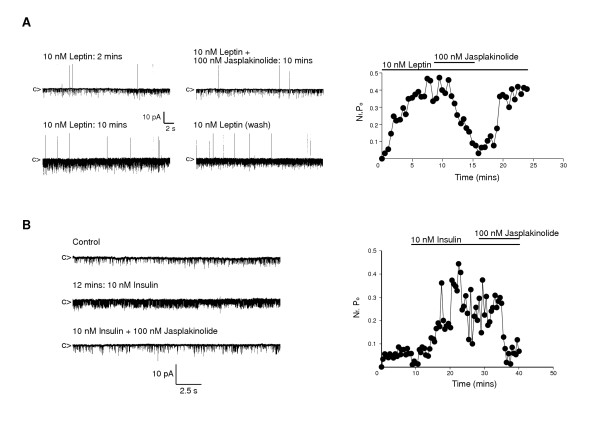
**Actin dynamics mediate leptin and insulin activation of K_ATP _***A*, representative cell-attached recording from an acutely isolated ARC neurone with leptin (10 nM) in the electrode solution. Following attainment of increased K_ATP _activity, bath application of jasplakinolide (100 nM) reversibly reduced channel activity. The corresponding diary plot for this experiment is shown. *B*, representative cell-attached recording from an ARC neurone. Bath application of insulin (10 nM) increased K_ATP _channel activity and subsequent bath addition of 100 nM jasplakinolide, concomitant with 10 nM insulin, reversibly inhibited the insulin stimulated K_ATP _activity. The corresponding diary plot is shown.

Previous studies have demonstrated that the phosphatidylinositol lipid second messenger, PtdIns(3,4,5)P_3 _activates K_ATP _channels in an insulin-secreting cell line when applied directly to the internal aspect of isolated patches [23]. However, activation of K_ATP _is probably not due to direct binding of the lipid to channel subunits, as the effect of PtdIns(3,4,5)P_3 _on K_ATP _was prevented by the presence of the actin stabilizing agent, phalloidin. Additionally, leptin-induced opening of this insulin-secreting cell K_ATP _channel was occluded when phalloidin was present in the cell interior [23]. Thus we examined whether adiposity hormone signalling in ARC GR neurones also requires actin remodelling in order to manifest a specific effector output, the activation of hypothalamic neurone K_ATP _channels. As our assessment of hormone activation of GR neurone K_ATP _channels uses cell-attached recordings, we used the membrane permeable actin stabilizing toxin, jasplakinolide to induce actin polymerization [24]. In preliminary experiments, jasplakinolide (100 nM) was demonstrated to have no effect when applied directly to isolated inside-out patches obtained from ARC neurones, under asymmetrical recording conditions, containing spontaneously active K_ATP _channels (n = 4; P > 0.5; data not shown). Cell-attached recordings with leptin (10 nM) present in the pipette solution, increased mean K_ATP _channel activity from 0.07 ± 0.02 to 0.44 ± 0.07 (n = 4; P < 0.01). Subsequent bath application of jasplakinolide (50 – 100 nM) reversed the leptin-induced K_ATP _activation (Figure 6A), with channel activity returning to 0.10 ± 0.02 (n = 4; P < 0.01) within 5–10 minutes, a level indistinguishable from pre-leptin controls (P > 0.5). In a second series of cell-attached recordings, bath application of insulin (0.1 – 10 nM) increased mean K_ATP _activity from 0.06 ± 0.01 to 0.64 ± 0.16 (n = 4; P < 0.01) and subsequent bath application of jasplakinolide (100 nM), in the continuous presence of insulin, reduced channel activity to 0.17 ± 0.06 (n = 4; P < 0.01) within 5–10 minutes (Figure 6B). The inhibition of leptin and insulin stimulated K_ATP _channel activity by jasplakinolide was reversible on washout of the toxin in 2/4 and 3/4 patches for leptin and insulin respectively.

### PI3K mediates leptin-induced actin filament reorganisation in GT1-7 cells

**Figure 7:**
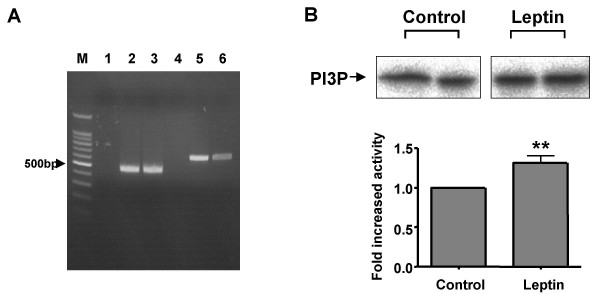
**Leptin stimulates PI3K activity in GT1-7 cells ***A*, expression of the leptin receptor mRNA in mouse GT1-7 cells. Lanes 1–3, RT-PCR detection of the common ObR isoform in hypothalamus (lane 2) and GT1-7 cells (lane 3), together with a negative control (lane 1). Lanes 4–6, RT-PCR detection of the ObRb isoform in the hypothalamus (lane 5) and GT1-7 cells (lane 6) together with a negative control (lane 4). Note the presence of PCR products of the appropriate sizes in GT1-7 and hypothalamus (465 bp ObR and 647 bp ObRb). *B*, PI3K activity associated with IRS-2 in GT1-7 cells stimulated with 50 nM leptin. PI3K activity was measured in immunoprecipitates and was quantitated using a Phosphoimager. Data are mean ± SEM for 4 experiments. ** P < 0.01.

**Figure 8:**
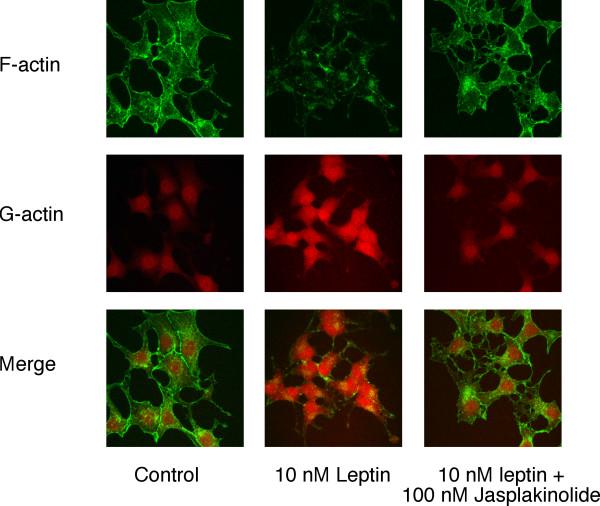
**Leptin disrupts cortical actin filaments in GT1-7 cells **Cultured GT1-7 cells were incubated in the absence and presence of leptin (10 nM) ± jasplakinolide (100 nM) for 30 minutes (jasplakinolide added 10 minutes prior to leptin). Following treatment cells were fixed and permeabilized, as described in the Methods, incubated with Alexa 488 conjugated phalloidin and Alexa 594 conjugated DNase I and subsequently processed for visualising F- and G-actin respectively by confocal microscopy.

**Figure 9:**
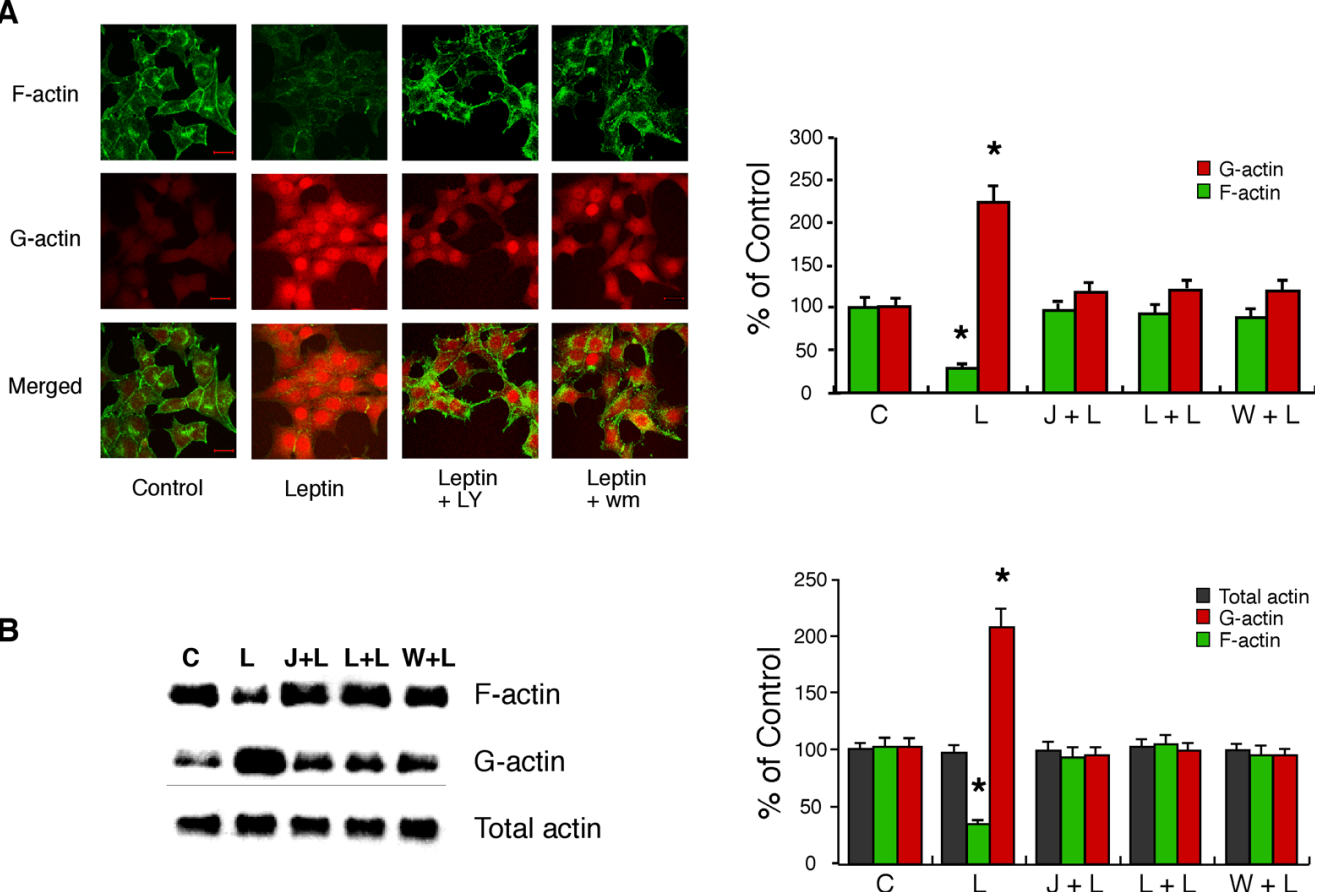
**PI3K mediates F-actin disruption in GT1-7 cells ***A*, Cultured GT1-7 cells were incubated in the absence and presence of leptin (10 nM) ± wortmannin (10 nM) or LY 294002 (10 μM) for 30 minutes. Following treatment cells were fixed and permeabilized, as described in the Methods, incubated with Alexa 488 conjugated phalloidin and Alexa 594 conjugated DNase I and subsequently processed for visualising F- and G-actin respectively by confocal microscopy. *B*, Plot of average alexa 488-phalloidin fluorescence intensity (green) and alexa 594-DNase 1 (red) in fixed cells treated with 10 nM leptin (L) alone or cells treated with leptin and jasplakinolide (100 nM; J + L), LY294002 (10 μM; L + L) or wortmannin (10 nM; W + L) relative to cells untreated (C) with drug (n = 8 separate experiments, with 8 cells measured under each condition for each experiment). *C*, GT1-7 cells were incubated with PBS only (C), 10 nM leptin (L) or leptin and jasplakinolide (100 nM; J + L), LY294002 (10 μM; L + L) or wortmannin (10 nM; W + L). Cells were treated to extract actin pools as described in Methods and equal amounts of pool lysate were subjected to SDS-PAGE and transferred to nitrocellulose membrane. The levels of actin were detected by immunoblotting with an actin monoclonal antibody. *D*, Plot of average Triton-X-100 soluble (G, red), Triton-X-100 insoluble (F, green) and total actin (gray) concentration from live cells, relative to control untreated cells (n = 4 separate experiments), for data as shown in *C*. Error bars indicate s.e.m. and * significance of P < 0.01.

**Figure 10:**
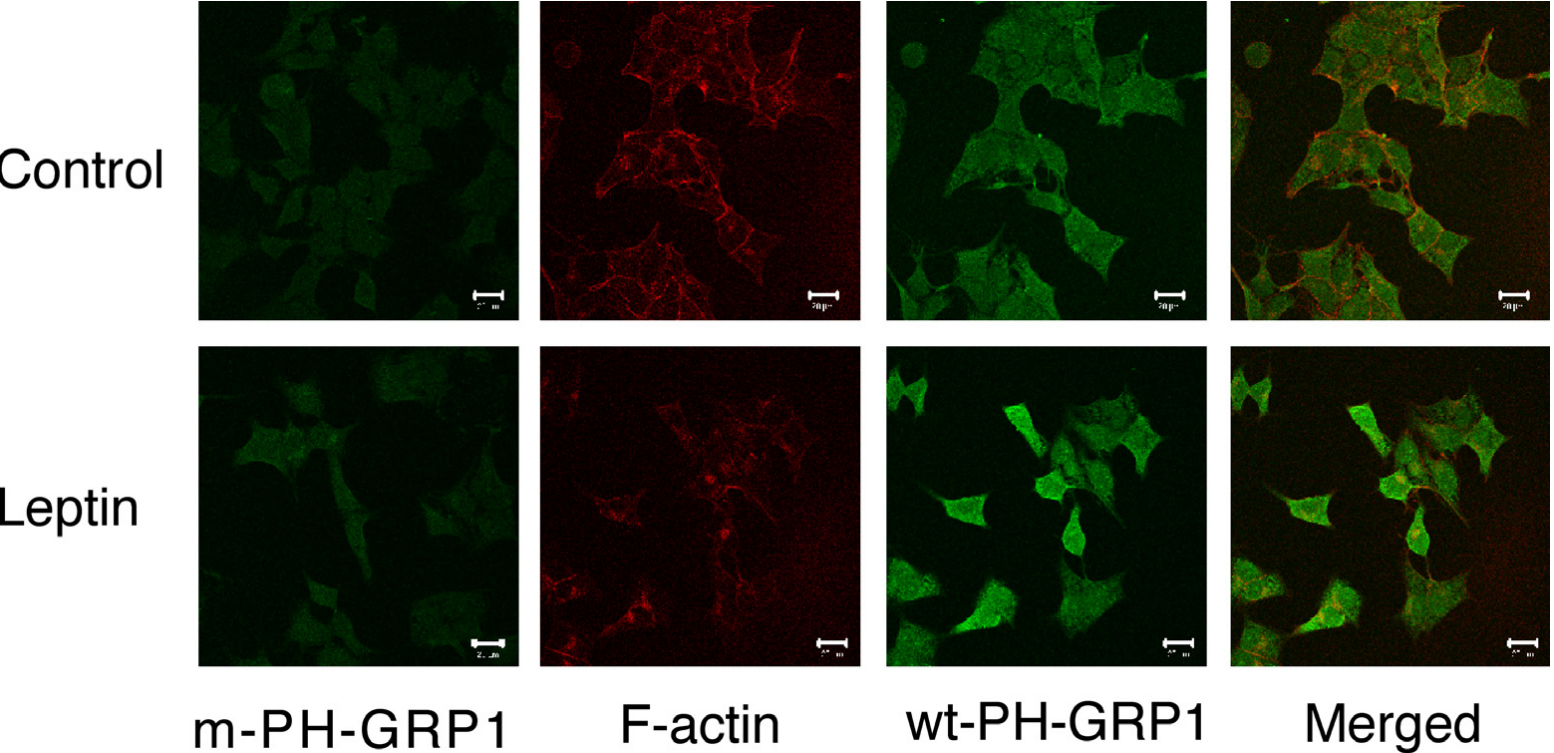
**Re-organisation of F-actin is associated with raised PtdIns(3,4,5)P_3 _levels in GT1-7 cells **Cultured GT1-7 cells were incubated in the absence and presence of 10 nM leptin for 20 minutes. Cells were fixed and permeabilized, as described in Methods, prior to incubation with rhodamine-conjugated phalloidin and wild type (wt) or K273A mutant (mt) PH-GRP1-GFP fusion protein for 60 minutes. Cells were subsequently processed for visualising GFP and rhodamine by confocal microscopy.

Preliminary experiments labelling rat ARC slices with rhodamine-conjugated phalloidin to stain for F-actin were unsatisfactory due to the overall high levels of staining in the slices and inability to distinguish clearly individual neurones and their responses to hormone stimulation. Similarly, use of freshly isolated ARC neurones was precluded as the phalloidin staining was inconsistent among individual neurones within a single preparation and between neuronal preparations. Thus we have used the mouse hypothalamic cell line, GT1-7, to demonstrate that leptin utilises a PI3K-dependent signalling cascade to modify cytoskeletal dynamics. A previous study indicated that GT1-7 cells express ObRb [25], although others do not concur with this conclusion [26]. Using RT-PCR, we detected the presence of leptin receptor mRNA in GT1-7 cells by amplification of a common extracellular domain of the mouse receptor. Further analysis using primers specific to ObRb, which contains a long cytosolic domain with the intracellular protein motifs required for signalling [10], demonstrates the presence of this receptor isoform in GT1-7 cells (Figure 7A). We have also used this hypothalamic cell line to examine whether leptin is capable of increasing PI3K activity directly. In response to 50 nM leptin, IRS-2-associated PI3K activity was modestly, but significantly, increased (Figure 7B). Thus, using native GT1-7 cells leptin (1–10 nM) induced a decrease in cortical F-actin as visualised by alexa 488 conjugated phalloidin staining, which was prevented or reversed by the presence of 100 nM jasplakinolide (n = 8; Figure 8 upper panels). As cellular cortical actin structure is determined by the dynamic equilibrium between F- and G-actin, a reduction in F-actin at the plasma membrane should be accompanied by a corresponding increase in the concentration of free G-actin in the cells [27]. Figure 8 (middle panel) demonstrates, using alexa 594 conjugated DNase I staining of the same cells, that leptin does indeed increase the concentration of G-actin and that this effect is also sensitive to the presence of jasplakinolide (n = 8). Indeed, dual staining of the GT1-7 cells demonstrates (Figure 8, lower panel) that leptin alteration of the cortical cytoskeleton is due to a concomitant increase in the content of G-actin at the expense of F-actin, and that this action is completely inhibited in the presence of jasplakinolide. The alteration in cytoskeletal dynamics by leptin is also PI3K dependent as shown in Figure 9A, where the presence of either 10 nM wortmannin (n = 13) or 10 μM LY294002 (n = 13) substantially reduced the ability of leptin to decrease the levels of F-actin and increase G-actin as assessed by phalloidin and DNase I staining respectively. This cell staining method of assessing leptin stimulated changes in actin dynamics was compared to direct quantitative analysis of actin. Live cells were treated with leptin (10 nM) ± jasplakinolide (100 nM) or LY294002 (10 μM) or wortmannin (10 nM) for 20 minutes, Triton-X-100 soluble (G) and insoluble (F) actin fractions separated and run on a gel [28]. Exposure of cells to leptin did not alter total cellular actin, whereas G-actin levels increased by 2 fold, at the expense of F-actin, the levels of which declined by 65% (n = 4; Figure 9C,9D). Thus, leptin induced a change in the G/F actin ratio from a control value of 0.54 to 3.17. These data correlate well with the change in fluorescence intensity observed in leptin-treated fixed cells (n = 8), where G-actin levels were also increased by 2 fold and F-actin decreased by 70% (Figure 9B). Exposure of cells to the F-actin stabilizing agent, jasplakinolide or the PI3K inhibitors, LY294002 or wortmannin prevented leptin from inducing F-actin disassembly as observed by either assay (Figure 9A,9B,9C,9D). In addition, protein overlay experiments using wild type PH-GRP1-GFP fusion protein binding to assess PtdIns(3,4,5)P_3 _levels in GT1-7 cells demonstrate that leptin increases PI3K activity concurrently with the re-organization of cortical actin in this cell line, with leptin stimulation inducing little change in the K273A mutant PH-GRP1-GFP binding to these cells (Figure 10).

## Discussion

### PI3K – a pivotal enzyme in ARC signalling

Previous studies have demonstrated that leptin applied *in vivo *stimulates hypothalamic ObRb to increase phosphorylation of the signalling protein intermediates STAT3 and MAPK and that both leptin and insulin increase hypothalamic PI3K activity [12,29]. Here we have applied hormones directly to ARC wedges isolated from hypothalamic slices to enable improved signal detection (with respect to amplitude and temporal resolution), localisation of signalling to the arcuate nucleus and to fix external conditions so that potential compensatory changes associated with *in vivo *studies are obviated. Exposure of ARC wedges to leptin or insulin induced rapid (≤1 minute) phosphorylation of MAPK (ERK1 & 2 subfamilies), STAT3 and the PI3K activity indicators, PKB and its downstream target GSK3. These hormone-induced increases in phosphorylation were transient in the majority of experiments, usually lasting 1–5 minutes at ~34°C with return to control levels of phosphorylation within 30 minutes. Such rapid recovery has also been noted in other studies [13,30] and may be due to activation of endogenous phosphatases such as PTP1B curtailing this acute signalling process [14,31]. The phosphorylation of MAPK is quite modest and at present there are few data which link this pathway directly with the actions of either insulin [32] or leptin [16] on energy homeostasis, although recently it has been shown that centrally driven insulin-mediated sympathoactivation of brown adipose tissue is MAPK-dependent [33].

As expected, exposure of ARC wedges to leptin induced an increase in tyrosine phosphorylated STAT3 [11,13,29,34]. However, unexpectedly insulin also induced an increase in tyrosine phosphorylation of STAT3 in ARC neurones. In a previous study [30]* in vivo *application of insulin (icv) demonstrated no such change, unless leptin was co-applied. The data reported here indicate that insulin *per se *is capable of increasing STAT3 phosphorylation, as no exogenous leptin was present or endogenous leptin likely to remain in the ARC sections following the extensive washes and incubations prior to stimulation. This difference may be due to an increased signal to noise delivered using ARC tissue over whole hypothalamus and that rapid transient signals are more readily detectable by this method. Both leptin and insulin rapidly increased the phosphorylation of PKB and its downstream effector GSK3 in a wortmannin and LY294002 sensitive manner, indicative of increased PI3K activity in ARC neurones, in agreement with previous *in vivo *studies [17,35].

However, our results did not demonstrate that either leptin or insulin induced a significant increase in IRS-2-associated PI3K activity measured directly in ARC tissue. This may be due to a low signal to noise ratio, as only a (unknown) proportion of cells would be expected to respond to the hormones in the ARC tissue block, and/or that hormone mediated increases in PI3K activity are limited to plasma membrane microdomains. This is supported by the very modest increase in PI3K activity detected in GT1-7 cells when stimulated by leptin. Although hypothalamic activation of PKB by insulin has been reported previously [17], these are the first reports that leptin increases PKB activity and that both hormones increase the phosphorylation of GSK3 in the ARC. The presence of the PI3K inhibitors, wortmannin or LY294002, also reduced the leptin and insulin driven increase in MAPK phosphorylation. The mechanism by which leptin and insulin cause phosphorylation of this protein is most likely through the Ras pathway, as this protein has been demonstrated to interact directly with the catalytic subunit of PI3K [36] and inhibitors of PI3K have been reported to inhibit insulin induced increased MAPK activity, for example in rat adipocytes [37]. The insulin mediated enhanced STAT3 tyrosine phosphorylation in an interesting observation that requires further examination. Although phosphorylation of tyrosine-705 on STAT3 is a prerequisite for dimerisation and translocation of STAT3 to the nucleus [38], phosphorylation of serine-727 may also be required for maximal activation of STAT3 DNA binding [39].

Interestingly one pathway candidate for phosphorylating serine-727 is the Ras/Raf/MEK signalling cascade, and indeed a recent study has demonstrated that leptin can induce S727 phosphorylation of STAT3 in a PD98059 dependent manner in macrophages, and this is required to produce full stimulation of STAT3 [40]. Insulin mediated serine phosphorylation of STAT3 has also been reported, using transfected Chinese hamster ovary cells, to be mediated by a MEK-dependent pathway [41]. A similar mechanism in hypothalamic neurones would indicate an inter-connection between the three identified signalling pathways activated by these hormones and an important effector molecule, STAT3. Studies are underway to examine this proposal.

The importance of STAT3 signalling to the central mechanisms that control energy homeostasis has recently been directly demonstrated by transgenic mouse studies. Using a 'knock-in' strategy to induce defective STAT3 binding to ObRb [42] or a 'knock-out' strategy to ablate STAT3 from some hypothalamic neurones [43], loss or reduction in hypothalamic STAT3 signalling initiates hyperphagia, increased body weight and adiposity with alterations in glucose homeostasis. Indeed, the JAK2-STAT3 and IRS2-PI3K signalling pathways are purported to underpin the genomic and acute or membrane functions of these signalling pathways respectively [12]. Clearly, further work is required to determine the exact signalling mechanisms controlling insulin stimulated STAT3 phosphorylation in hypothalamic neurones.

### Leptin and insulin signalling to K_ATP _channels

Leptin and insulin cause inhibition, by hyperpolarization through activation of a sulphonylurea-sensitive K^+ ^conductance, of a subset of hypothalamic neurones, defined by their acute sensitivity to changes in external glucose concentration, termed GR neurones [6,7,19]. Single channel recordings from acutely isolated ARC neurones demonstrate that both hormones activate the same K^+ ^channel, the sulphonylurea-sensitive large conductance K_ATP _channel. This action is rapid and independent of transcriptional events, so most likely is mediated by MAPK or PI3K signalling. Pharmacological inhibition of the MAPK pathway with PD98059 did not reverse leptin (as shown above) or insulin [7] stimulated K_ATP _activity, abrogating this pathway from causing the hyperpolarising response. In contrast, inhibition of PI3K with either wortmannin or LY294002, reversed both leptin (as shown above) and insulin [7] raised K_ATP _activity. Furthermore, use of the fusion protein PH-GRP1-GFP as a specific detector of PtdIns(3,4,5)P_3 _in isolated neurones also demonstrated that both hormones rapidly increase the cellular content of this PI3K lipid product in a sub-population of neurones. These results are consistent with class 1 PI3K [44] acting as a point of convergence for leptin and insulin signal transduction pathways to K_ATP _channels in GR neurones. The functional significance of PI3K in the control of energy balance has been demonstrated by *in vivo *studies, which show that leptin [16] and insulin [17] stimulate IRS2-associated PI3K activity in the hypothalamus and pharmacological inhibition, using wortmannin and LY294002, of hypothalamic PI3K activity prevents the anorectic actions of icv leptin or insulin, whereas the MAPK inhibitor PD98059 had no effect on leptin driven attenuation of food intake [16].

### Remodelling of cortical actin filaments as a leptin and insulin signalling event

Leptin and insulin stimulated K_ATP _activity in isolated ARC neurones was also reversed, within 5–10 minutes, by the marine sponge toxin, jasplakinolide. This toxin binds to F-actin with high affinity, resulting in its stabilization and prevention of depolymerization to its monomer G-actin [24]. These data indicate that the adiposity hormones require actin filament depolymerization for K_ATP _activation to occur. Such a mechanism is supported by reports that agents, which promote actin depolymerization, activate K_ATP _channels in cardiac myocytes [45,46] and the insulin-secreting cell line, CRI-G1 [47]. Furthermore, in this latter study leptin stimulated K_ATP _channel activity was also shown to depend on actin filament depolymerization. Insulin is also well documented to cause actin filament re-organization in peripheral cells associated with various functional outputs, which depend on PI3K activity, including metabolic and mitogenic effects [48]. The reversal of hormone-stimulated K_ATP _activity by jasplakinolide was faster (5–10 minutes) than for the PI3K inhibitors (15–20 minutes). This temporal difference suggests that the site of jasplakinolide action is downstream from the PI3K signal transduction pathway to K_ATP _channels.

However, alteration of the cellular cortical actin structure is inferred through the use of natural agents like jasplakinolide. In order to verify directly that hormone-driven structural re-arrangements did occur we decided to use the hypothalamic cell line, GT1-7, as preliminary experiments using freshly isolated neurones did not produce reliable and reproducible data due to the presence of dead and dying cells showing as false positives for hormone induced actin depolymerization. Use of this cell line also obviated any problems with identification of ObRb containing neurones and neuronal subtypes in slices. RT-PCR analysis indicates that this cell line does express the main signalling form of the leptin receptor and analysis of PI3K activity shows functional coupling of this receptor to this signalling pathway. We have shown, by cell staining of fixed cells and, independently by analysis of cellular G- and F-actin concentration from live cells, that leptin disrupts cortical actin structure by disturbing the processes that maintain the equilibrium between F-actin and G-actin, in the direction of depolymerization to G-actin. This effect of leptin was completely inhibited by the presence of either jasplakinolide or the PI3K inhibitors. In addition, there is a good temporal and spatial association between PtdIns(3,4,5)P_3 _production, as determined by PH-GRP1-GFP binding, and actin filament depolymerization. Thus, leptin and insulin signalling in, at least some sub-groups of hypothalamic neurones maintains a close parallel with leptin signalling in insulin-secreting cells, where it has been reported that leptin increases K_ATP _activity by a PI3K-dependent cortical actin re-arrangement [47].

## Conclusions

The effect of leptin and insulin on the phosphorylation status of various cellular signalling intermediates and on K_ATP _channel activation in arcuate neurones indicates that both hormones activate the same signalling cascades, and can produce common outputs. The sensitivity of both K_ATP _opening and the phosphorylation of certain intermediates to PI3K inhibition is significant as this enzyme has been previously demonstrated to play an important role in leptin and insulin mediated energy homeostasis control. Furthermore it is interesting that leptin and insulin induce rapid phosphorylation of MAPK and STAT3 as these data support the view that these hormones may influence genomic and membrane neuronal outputs by common mechanisms. The inhibition of leptin and insulin stimulation of K_ATP _channel opening of arcuate neurones by jasplakinolide suggests a role for cytoskeletal dynamics in modulation of membrane events such as neuronal hyperpolarization. This hypothesis is further strengthened by the finding that leptin induces actin filament depolymerization in a mouse hypothalamic cell line, which is PI3K dependent, demonstrating that this cell line may be a useful model for further analysis of leptin signalling mechanisms in hypothalamic neurones.

## Methods

### Preparation of hypothalamic lysates and immunoblots

Male Sprague-Dawley rats (50–100 g) were killed by cervical dislocation in accordance with Schedule 1 of the UK Government Animals (Scientific Procedures) Act (1986). The brain was rapidly transferred to ice-cold aCSF solution, containing (in mM): 128 NaCl, 5 KCl, 1.2 NaH_2_PO_4_, 26 NaHCO_3_, 1.2 CaCl_2_, 2.4 MgSO_4_, and 10 glucose, equilibrated with 95% O_2_, 5% CO_2 _to give a pH of 7.4. The tissue was maintained in ice-cold aCSF whilst horizontal 400 μm coronal brain slices were prepared using a Vibratome (Intracel, Royston, Herts. UK). Slices containing the ARC were incubated in aCSF at room temperature for 20 minutes, and then at 33–35°C for 1 hour. Hypothalamic wedges, predominantly containing the ARC were cut, and these were incubated in aCSF ± hormones and/or kinase inhibitors (10 mls) for the required time. The reaction was stopped by the addition of 2 ml of cold lysis buffer containing (in mM) 100 NaCl, 10 NaF, 25 Tris HCl, 10 NaPP_i_, 5 EGTA, 1 EDTA, 1 Na_3_VO_4_, 1 Benzamidine, 0.1 PMSF, 0.1% (v/v) mercaptoethanol, 1% Tritron X-100 (v/v) and 92 mg ml^-1 ^sucrose. The tissue was homogenised on ice, the lysate sonicated for two 10 s periods and then centrifuged for 10 minutes at 12000 rpm at 4°C. The supernatant was retained and the pellet discarded. The protein content of the clarified lysate was determined by the method of Bradford [49]. Proteins (10 μg) were separated by SDS-PAGE, and subsequently transferred to nitrocellulose membranes. Membranes were incubated in blocking buffer (10% non-fat dried milk in TBST (20 mM Tris HCl, 150 mM NaCl, 0.5% Tween, pH 7.4)) for 1 hour at room temperature following which phospho-specific p44/p42 MAPK (Thr202/Tyr204), phospho-specific STAT3 (Tyr705), phospho-specific GSK-3α/β(Ser21/9), phospho-specific PKB (Thr308) and PKB (all polyclonal and used at 1:1000) antibodies were applied overnight at 4°C with gentle shaking. All antibodies were obtained from Cell Signalling Technology Inc. The membranes were washed four times with TBST and incubated for 1 hour at room temperature with horseradish peroxidase conjugated Goat anti-Rabbit IgG (1:5000). After further washing with TBST, total amount of specific protein was visualised by enhanced chemiluminescence detection as described by the manufacturer (NEN Life Science Products). Immunoreactive bands were scanned and quantified using AIDA software. As an internal control, the membranes were immunoblotted with a monoclonal anti β-actin antibody (Sigma: used at 1:5000) or with the PKB antibody. The values for proteins were normalized with respect to the internal control to account for variations in gel loading.

### Determination of PI 3-kinase activity

Cell and tissue lysates were made as described. The immunoprecipitation and PI3K activity assay were carried out as previously described [50]. Briefly, frozen samples were thawed before centrifugation to remove precipitated material. 10 μl Protein-G-Sepharose beads pre-coupled to 5 μg anti-IRS2 antibody (Upstate Biotechnology) was used to immunoprecipitate PI3K activity from ~0.5 mg cell lysate. The immunoprecipitated material was washed once with ice cold lysis buffer and three times with ice cold assay buffer, both of which were freshly supplemented with protease inhibitors, reducing agent and sodium vanadate as described [51]. Washed beads were re-suspended in 40 μl assay buffer supplemented with 1 μM unlabelled ATP, 25 μCi/assay radiolabelled ATP and phosphatidylinositol/phosphatidylethanolamine vesicles (final concentration of each lipid 100 μM). Samples were incubated at 37°C for 30 mins and the reaction was stopped by addition of 0.6 ml methanol/chloroform/12 M HCl (80:40:1, v/v), 0.2 ml chloroform and 0.32 ml 0.1 M HCl. Samples were processed and PtdIns(3)P separated from contaminating materials by thin layer chromatography (TLC) as previously described [51]. Bands corresponding to [^32^P]PtdIns(3)P were located using a phosphorimager (Fuji FLA 5000) and analyzed with AIDA software.

### Preparation of acutely isolated ARC neurones and electrophysiology

Coronal slices containing the medial hypothalamus were obtained (as described above) and sections containing the ARC were removed. The sections were transferred to 5 ml aCSF containing 1 mg ml^-1 ^protease XIV (Sigma-Aldrich, Dorset, U.K) and incubated for 1 hour at room temperature. The aCSF was continuously gassed with 95% O_2_: 5% CO_2 _for the entire incubation period. Sections were removed and washed in 50 ml aCSF five times prior to re-suspension in 5 ml normal saline containing (in mM): 135 NaCl, 5 KCl, 1 MgCl_2_, 1 CaCl_2_, 10 HEPES, 3 glucose, pH 7.4. Sections were sequentially triturated with fire polished Pasteur pipettes with decreasing tip size. The cell suspension was evenly distributed onto concanavalin A (Sigma-Aldrich) pre-treated 35 mm diameter culture dishes. The culture dishes were left for 15–20 minutes allowing cell adhesion prior to use.

Cell-attached single channel currents were recorded from single neurones at room temperature, using an Axopatch 200B amplifier (Axon Instruments, Foster City, CA USA). Patch pipettes were prepared from thick walled borosilicate glass and had open tip resistances of 8 – 15 MΩ when filled with high K^+ ^solution containing (in mM) 140 KCl, 1 MgCl_2_, 1 CaCl_2_, 10 HEPES, pH 7.2. This solution was used in order to allow easy identification of K^+ ^currents in the cell-attached configuration [19]. All cell-attached recordings were made in the presence of normal saline, with no applied pipette potential, thus utilizing the cell membrane potential to drive current flow (with inward current shown as downward deflections). Single channel recordings from inside-out patches isolated from ARC neurones were made either under asymmetrical conditions, in the presence of normal saline, or under symmetrical K^+ ^conditions with the intracellular aspect of the membrane exposed to a bathing solution containing (in mM): 140 KCl, 1 MgCl_2_, 2.7 CaCl_2_, EGTA 10 (free Ca^2+ ^of 100 nM), HEPES 10, pH 7.2.

Data were recorded onto digital audio-tape using a Biologic DTR 1200 recorder and analysed off-line. Pre-recorded data were transferred via a Digidata 1200 interface into a PC, digitised at 10 kHz and measured using the PCLAMP6 software, Fetchan 6. The mean current (I) and single channel amplitude (i) were determined for recordings ranging in duration from 30 s to 120 s and channel activity (N_f_.P_o_) determined as described previously [52], where N_f _is the number of functional channels and P_o _is the open probability. Drug effects were measured by comparison of N_f_.P_o _from individual patches in the presence and absence of the drug. Data for a given set of experiments were normalised and statistical significance determined by employing the Students *t*-test for unpaired data. Results are presented as mean ± SEM and the number of experiments denoted by 'n.'

### Leptin receptor mRNA expression

Reverse transcription was performed in a 20 μl reaction containing 1 × First Strand Buffer, 1 mM DTT, 0.5 mM of dNTP, 0.5 μg anchored oligo(dT)18, 4 μg RNA and 1 μl (200 U) M-MLV Reverse Transcriptase (Gibco), at 25°C for 5 minutes, 42°C for 60 minutes, 70°C for 15 minutes and stored at -20°C. After RT, a 2 μl aliquot of the reaction was added to 48 μl of PCR mix. The mix containing 1 × PCR buffer, 2.5 mM MgCl_2_, 0.5 mM PCR nucleotide mix, 1 μM each of the gene specific primers (mObRcom F:ggaatgagcaaggtcaaaa; mObRcom R:gtgacttccatatgcaaacc; mObRb F:tcttctggagcctgaacccatttc; mObRb R:ttctcaccagaggtccctaaact; ref [53]) and 5 units of Taq DNA polymerase (Promega). PCR was performed using the following profile: 94°C for 5 minutes, 25 cycles at 94°C for 30 seconds, 55°C for 30 seconds, 72°C for 30 seconds, with a final extension at 72°C for 7 minutes.

### GT1-7 cell culture, staining and actin analysis

The mouse hypothalamic cell line GT1-7 [54] was grown in Dulbecco's modified Eagle's medium supplemented with 10% fetal calf serum (Sigma), 1 mM L-glutamine and 1% penicillin-streptomycin at 37°C in a humidified atmosphere of 95% air and 5% CO_2_. Cells were passaged every 3–4 days, plated on poly-L-lysine (Sigma) coated glass coverslips in 3.5 cm Petri dishes and used 1–2 days after plating. Cells were treated with 10 μM LY294002 or 10 nM wortmannin or 100 nM jasplakinolide (all Sigma) in normal saline for 10 minutes, prior to a challenge with 10 nM leptin (or saline), in the continuous presence of inhibitor, for 20 or 60 minutes before fixing. GT1-7 cells were fixed in 4% methanol-free formaldehyde in cytoskeletal buffer (10 mM MES, 3 mM MgCl2, 138 mM KCl, 2 mM EGTA, pH 6.1) with 0.32 M sucrose for 30 minutes [28]. They were then washed in phosphate-buffered saline (PBS), permeabilised in PBS/0.5% Triton X-100 for 10 minutes, rinsed in PBS, blocked with 20% goat serum (Sigma) for 30 minutes, rinsed in PBS and incubated with rhodamine conjugated phalloidin, or 2 μg ml^-1 ^Alexa 594-DNase I and 2 U ml^-1 ^Alexa 488-phalloidin (all Molecular Probes) for 90–120 minutes, rinsed in PBS, and mounted on coverslips. Cells were observed with a 63X oil objective and images acquired using a laser-scanning confocal microscope (Zeiss LSM 510), under identical conditions with randomly selected regions of each coverslip. For quantitative analysis of G- and F-actin cellular pools, we used a direct method to partition the actin pools from live cells [28]. In brief, equal cell numbers were added to 3.5 cm culture dishes and cells grown to 80% confluence. The Triton-X-100 soluble (G-actin) pool was isolated first, by incubating cells for 5 minutes at room temperature with 1 ml PBS containing 1% Triton-X-100, protease inhibitors and 1 μg ml^-1 ^phalloidin (to prevent filament dissociation). Cells were then washed with PBS, and the Triton-X-100 insoluble pool (F-actin) prepared by addition of 1 ml of PBS lysis buffer, containing 1% Triton-X-100, protease inhibitors, 2% SDS and 1 μg ml^-1 ^phalloidin for 5 minutes prior to harvesting cells from dishes. For determination of total actin, cells were exposed to the second step only. Each cellular pool was passed through a 25 gauge needle and total protein concentration determined, before equal amounts of protein were loaded onto SDS-PAGE gels, and actin detected using an actin monoclonal antibody (Chemicon). Quantitative measurements of G- and F-actin in fixed cells were made using Velocity software (Improvision), where individual cell total fluorescence, normalized to cell area, was determined and background fluorescence subtracted. Average fluorescence intensity was calculated for 8 cells in each experiment, and expressed relative to control (non-drug exposed cells). Actin bands on gels were quantified by densitometry, where total density was determined with respect to constant area, background subtracted and average relative band density calculated.

### PH-GRP1-GFP fusion protein overlays

Following stimulation with hormone for 10–20 minutes, acutely isolated neurones (room temperature) or GT1-7 cells (37°C) were fixed at room temperature with 2–4% paraformaldehyde for 15 and 30 mins, respectively. Cells were permeabilized by washing with 0.05% PBS-Tween 20 (PBS-T; x2 for 10 mins). Non-specific binding was minimised by blocking with 3% BSA for 1 hour at room temperature. Cells were subsequently washed with 0.05% PBS-T prior to incubation with wild type PH-GRP1-GFP or K273A mutant PH-GRP1-GFP (50 μg ml^-1^) fusion protein for 1 hour at room temperature, and images acquired by confocal microscopy.

## List of abbreviations

ACSF, artificial cerebrospinal fluid; AgRP, agouti-related protein; ARC, arcuate nucleus; NPY, neuropeptide Y; POMC, proopiomelanocortin; K_ATP_, ATP-sensitive potassium channel; BBB, blood-brain-barrier; DNase I, deoxyribonuclease I; F-actin, filamentous actin; G-actin, globular actin; GFP, green fluorescent protein; GR neurone, glucose-responsive neurone; GRP1, general receptor for phosphoinositides-1; GSK3, glycogen synthase kinase 3; IRS2, insulin receptor substrate 2; JAK2, janus kinase 2; MAPK, mitogen-activated protein kinase; MEK, MAPK kinase; ObR, leptin receptor; PH domain, pleckstrin homology domain; PI3K, phosphoinositide 3-kinase; PKB, protein kinase B; PtdIns(3,4,5)P_3_, phosphatidylinositol 3,4,5-trisphosphate; STAT3, signal transducer and activator of transcription 3;

## Authors' contributions

SM carried out the electrophysiology studies, participated in the western blot and protein overlay experiments. HL carried out the arcuate western blot experiments and PI3K activity measurements. KN carried out the actin imaging and actin quantitative analysis experiments. EA contributed to the actin imaging experiments, western blots and participated in the protein overlay experiments. LB performed all tissue culture and participated in the western blot experiments. AG made the fusion proteins and participated in the design of the overlay experiments. CS participated in the design and implementation of the western blot experiments. MA conceived of the study, participated in its design and co-ordination and drafted the manuscript. All authors read and approved the final manuscript.
